# Predictive value of the number of frozen blastocysts in live birth rates of the transferred fresh embryos

**DOI:** 10.1186/s13048-021-00838-5

**Published:** 2021-06-26

**Authors:** Jianyuan Song, Cuicui Duan, Wangyu Cai, Jian Xu

**Affiliations:** 1 The Fourth Affiliated Hospital, Zhejiang University School of Medicine, No.1 Shang Cheng Avenue, Yiwu, 322000 Zhejiang China; 2 Women’s Hospital School Of Medicine, Zhejiang University, No.1 Xue Shi Road, Hangzhou, 310006 Zhejiang China

**Keywords:** Embryo quality, Follicle growth, Blastocyst, Ovarian response, Live birth

## Abstract

**Background:**

Blastocyst development by extended culture in vitro allows the embryos to ‘select’ themselves, thus successful growth to the blastocyst stage is a reflection of the developmental competence of cleavage stage embryos in a cohort. The study aims to determine whether the number of frozen blastocysts is associated with live birth rates of the transferred fresh embryos.

**Method:**

The retrospective study included 8676 cycles of first fresh embryo transfer from January 2016 to June 2019 at a fertility center of a university hospital. The patients with ≥ 10 oocytes retrieved were divided into three groups according to the number of frozen blastocysts: 0 (group 1), 1–2 (group 2), and ≥ 3 (group 3). The primary outcome measure was the live birth. The secondary outcome measures included clinical pregnancy rates and implantation rates. Logistic regression analysis was also performed.

**Results:**

Live birth rates in patients with ≥ 3 and 1–2 frozen blastocysts were 47.6% and 46.1%, respectively, which were significantly higher than that in patients without blastocyst (36.0%). The clinical pregnancy rate in group 3 was 65.1%, which was also significantly higher than the other two groups (47.0% and 59.2%). The implantation rates were 35.5%, 47.6%, and 56.0% in the three groups, respectively (*P *< 0.001). Compared with groups of frozen blastocysts, 0 frozen blastocyst yielded a lower rate of live birth (the adjusted odds ratio: 0.526, 95% CI: 0.469, 0.612).

**Conclusion:**

In patients with optimal ovarian response that retrieved ≥ 10 oocytes, fresh embryos transfer followed by having blastocysts frozen is a strong indicator of pregnancy achievement, but the number of frozen blastocysts (if not = 0) has limited value in predicting live birth rates.

## Introduction

As an independent factor for the success of in vitro fertilization (IVF) treatment, embryo quality is mainly evaluated by the morphological score including cell size, cell number, symmetry, percentage of fragments, defects of cytoplasm, and multinucleation [1–4]. However, the facts that ideal pregnancy rate were not always observed from embryos with even the highest possible score call in question the predictive value of morphological criteria [5, 6].

Thanks to controlled ovarian hyperstimulation (COH) for recruiting more follicles, patients usually have more than two utilizable embryos. Apart from investigating the relationships between the quality of the transferred embryos and pregnancy outcomes, many studies have suggested that the quality of un-transferred or supernumerary embryos may be positively correlated with implantation rates [7–10]. But these researches focused only on the number or quality of cleavage stage embryos. A retrospective cohort study of 655 single-blastocyst transfers concluded that the number of supernumerary vitrified blastocysts correlated positively with live birth in blastocyst transfers [11]. But we are not aware of any data examining the correlation between the number of supernumerary blastocysts and live birth rates in fresh embryos transfers.

Besides being transferred in fresh cycles, the extra embryos will undergo several procedures after fertilization such as cleavage stage, extended period of culture in vitro, blastocyst formation, and cryostorage. Not all remaining embryos are able to be frozen at the blastocyst stage for the future cycles. Even the post-fertilization circumstances are different, the dynamic process of embryos cultured in vitro can reflect the average developmental competence of embryos which were retrieved and fertilized in the same cohort. However, there is little evidence on the association between the number of supernumerary frozen blastocysts and the live birth of the transferred cleavage stage embryos.

It is noteworthy that the predicative value of the number of frozen blastocyst on live birth rates might be limited in sub-optimal ovarian responders because of its unstable statistical results. The objective of the present study was to determine whether the number of frozen blastocysts was associated with live birth rates of the transferred embryos in a cohort in patients with optimal ovarian response. To obtain clear conclusions, the patients with ≥ 10 oocytes retrieved were divided into three groups according to the number of frozen blastocysts: 0 (group 1), 1–2 (group 2), and ≥ 3 (group 3). All patients underwent their first IVF/ intracytoplasmic sperm injection (ICSI) cycles with good quality embryos transferred. Clinical outcomes were compared among the three groups.

## Materials and methods

### Study design and participants

This is a retrospective study of IVF/ICSI with GnRH agonist treatment at a fertility center of a university hospital from January 2016 to June 2019. The inclusion criteria included the following: (1) patients with the first stimulation cycle; (2) cycles with GnRH agonist protocol; (3) patients undergoing the fresh embryo transfer; (4) patients with ≥ 10 oocytes retrieved. The exclusion criteria was: (1) egg donor; (2) preimplantation genetic diagnosis; (3) cycles where no oocytes were retrieved or all embryos were frozen.

### Stimulation protocols and definition of groups

In two ovarian stimulation protocols for IVF treatment in our study, pituitary down-regulation was initiated with 0.1 mg GnRH agonist (Diphereline; Beaufour Ipsen, France) from the follicular phase in the short protocol or mid-luteal phase in the long protocol. Then the recombinant human follicle stimulating (rhFSH) (Gonal-F; Serono, Switzerland) ranging from 75 to 300 IU was injected for ovarian stimulation and the dosage was adjusted by maternal age, antral follicle count (AFC) score, and weight. When 2 leading follicles reached a mean diameter of 18 mm or 3 leading follicles reached 17 mm, HCG 10,000 IU (HCG, Livzon Pharmaceutical Group Inc., China) was administered to trigger ovulation. Transvaginal oocyte retrieval was performed 34–36 h after HCG administration. Oocytes were fertilized using standard IVF or ICSI. Unless the quality of the embryo was very poor (> 50% fragments or three or fewer cells on day 3, embryo transfer Cancelled), usually one or two best-quality embryos were transferred on day 3. The additional good-quality embryos were continuously cultured in G1 culture medium (Vitrolife, Sweden) which was supplemented with 5 mg/mL human serum albumin until the blastocyst stage and then cryopreserved through vitrification for the subsequent frozen-thawed cycles. All incubations were done under an atmosphere of 5% O_2_, 6% CO_2_, 89% N_2_ and 95% humidity at 37 °C in 750 μL of medium in Nunclon Delta Treated 4-Well IVF Dishs (Thermoscientific, USA).

The number of frozen blastocyst was counted and divided into three groups: 0 (group 1), 1–2 (group 2), and ≥ 3 (group 3).

### Definition of clinical outcomes

The primary outcome measure was the live birth rate. The secondary outcomes included the clinical pregnancy rate, implantation rate, and miscarriage rate. Live birth rate was classified as delivery of any viable infant after 24 weeks. Clinical pregnancy was identified with the presence of an intrauterine gestational sac with fetal cardiac activity on transvaginal ultrasound two to three weeks after a positive pregnancy test. Implantation rate reflects the number of gestational sacs divided by the number of embryos transferred. Miscarriage rate was defined as pregnancy loss before 24 weeks.

### Statistical analysis

**Fig. 1:**
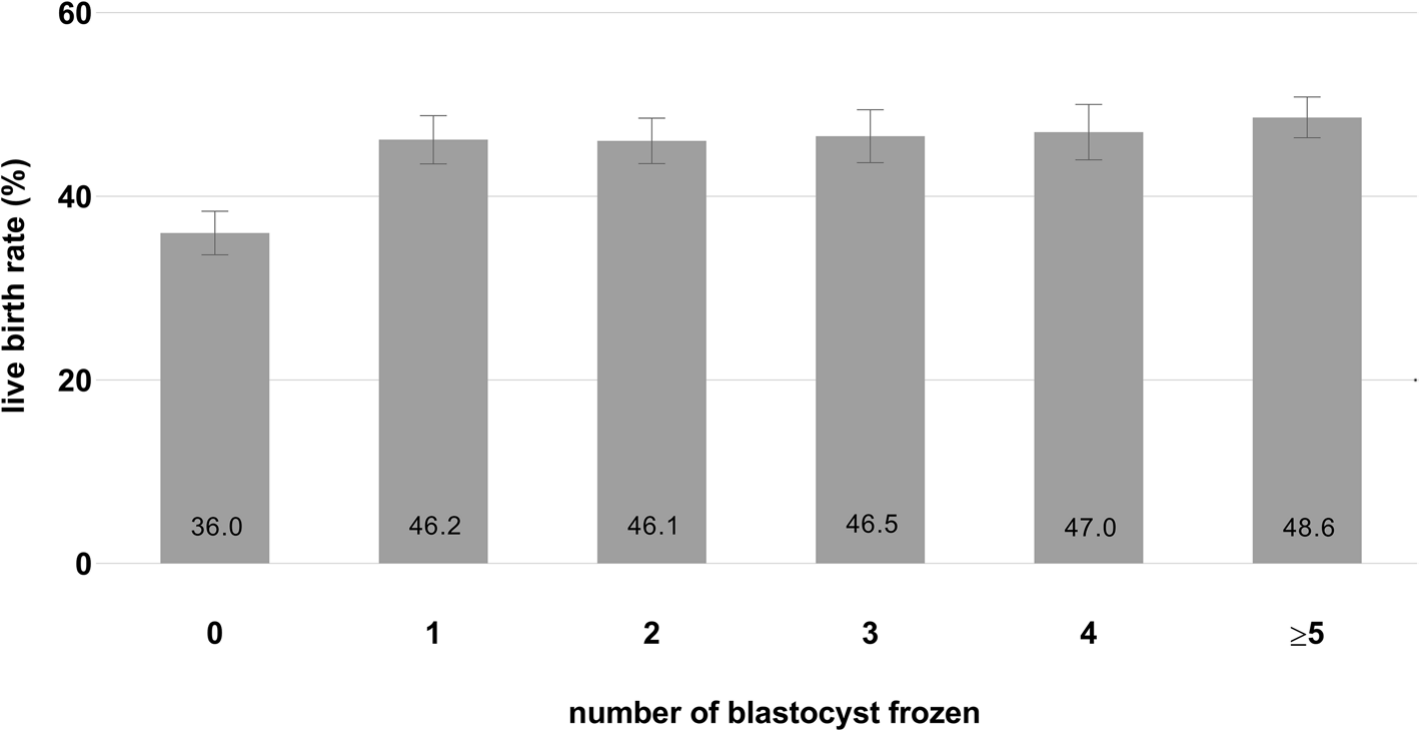
Live birth rates by the number of frozen blastocysts. The error bars show the 95% CIs. The number of patients with blastocyst of 0, 1, 2, 3, 4, and ≥ 5 was 1561 (18.0%), 1386 (16.0%), 1557 (17.9%), 1160 (13.4%), 1053 (12.1%), and 1959 (22.6%), respectively

Categorical data were expressed as number and/or percentage and were analyzed using Pearson's chi-squared. Continuous variables were identified for normal distribution by Kolmogorov–Smirnov test. Normally distributed data were expressed as mean ± standard deviation (SD) and were analyzed using analysis of variance (ANOVA). Non-normally distributed data were expressed as medians (interquartile ranges) (for) and were analyzed using Kruskal–Wallis test. Live birth rates were described by frozen blastocysts number of 0, 1, 2, 3, 4, ≥ 5 in Fig. 1.

Clinical outcomes per cycle were compared among the three groups with a Chi-square test. Then, a series of multivariate logistic regression was performed to assess whether the frozen blastocysts number was correlated with the live birth. Therefore, the live birth rate was adjusted according to female age, years of infertility, follicle stimulating hormone (FSH), antral follicle count (AFC), body mass index (BMI), type of infertility, the number of embryos transferred, endometrial thickness, type of treatment (IVF or ICSI). The results are given in terms of adjusted odds ratio (OR) and 95% confidence interval (CI).

*P *< 0.05 was considered statistically significant. All statistical analyses were carried out using the Statistical Package for Social science version 20.0 (SPSS, Inc.).

## Results

Division of 8676 patients according to the number of blastocyst frozen yielded 1,561 cycles in group 1 (0), 2,943 cycles in group 2 (1–2), and 4,172 cycles in group 3 (≥ 3) (Table 1).

**Table 1:** Demographic characteristics of the patients

	**Number of frozen blastocysts**			
	**0**	**1–2**	** ≥ 3**	***P***
Number of patients	1561	2943	4172	
Average number of frozen blastocysts	0	1.5 ± 0.5	4.9 ± 1.5	
Age (years)	29.9 ± 3.9	29.9 ± 3.6	29.7 ± 3.5	0.077
Years of infertility	3.0 (1.0, 18.0)	3.0 (1.0, 19.0)	3.0 (1.0, 19.0)	< 0.001
AMH (ng/mL)	4.9 (0.2, 18.0)	5.1 (0.1, 48.6)	5.7 (0.7, 34.5)	< 0.001
FSH (mIU/mL)	7.2 ± 1.8	7.2 ± 1.6	7.2 ± 1.8	0.359
AFC	15.3 ± 6.0	15.5 ± 5.9	16.4 ± 5.9	< 0.001
BMI	22.0 ± 3.0	22.0 ± 3.0	21.9 ± 3.0	0.100
Primary infertility	1038 (66.5%)	1948 (66.2%)	2757 (66.1%)	0.958
Estradiol (pg/mL)	2669.0 (433.9, 6500.0)	2705.0 (194.5, 6600.0)	2830.0 (434.0, 6880.0)	< 0.001
Progesterone (ng/mL)	0.8 ± 0.3	0.8 ± 0.3	0.8 ± 0.3	0.069
LH (mIU/mL)	1.1 (0.1, 7.2)	1.2 (0.1, 6.2)	1.2 (0.1, 5.1)	0.930
Endometrial thickness	12.3 ± 2.6	12.0 ± 2.6	12.0 ± 2.6	0.066
Number of oocytes retrieved	13.0 ± 3.5	13.5 ± 3.4	15.3 ± 3.8	< 0.001
Type of treatment				< 0.001
IVF	816	1790	2963	
ICSI	745	1153	1209	
Number of 2PN	5.7 ± 2.9	7.6 ± 2.8	10.2 ± 3.1	< 0.001
Blastocyst formation rate (%)^a^	30.8	44.8	70.1	< 0.001
Number of embryo transfer				< 0.001
1	713	1456	2756	
2	848	1487	1416	
OHSS rate (%)	1.8	1.9	2.1	0.751

Notes: Data are expressed as mean ± SD or medians (interquartile ranges) or number (percentage)

*AMH* Anti-Mullerian hormone, *FSH* Follicle stimulating hormone, *AFC* Antral follicle count, *BMI* Body mass index, *LH* Luteinizing hormone, *PN* Two visualized pronuclei, *IVF* In vitro fertilization and embryo transfer, *ICSI* Intracytoplasmic sperm injection, *OHSS* Ovarian hyperstimulation syndrome

^a^ Blastocyst formation rate: number of blastocysts formed per 2PN cultured

### Baseline characteristics and outcomes of COH

Characteristics of patients and ovarian stimulation procedures were displayed in Table 1. The average age of women enrolled in the three groups were 29.9 ± 3.9 years (mean ± SD), 29.9 ± 3.6 years, and 29.7 ± 3.5 years, respectively (*P *= 0.077). Medians (interquartile ranges) of the duration of infertility of women in the three groups were 3.0 (1.0–18.0), 3.0 (1.0–19.0), and 3.0 (1.0–19.0) years, respectively (*P *< 0.001). Women with more frozen blastocysts had longer higher AMH (medians 4.1 vs 5.1 vs 5.7 ng/mL, respectively, *P *< 0.001). As expected, more oocytes retrieved (13.0 ± 3.5, 13.5 ± 3.4, and 15.3 ± 3.8, *P *< 0.001) and number of two visualised pronuclei (2PN) (5.7 ± 2.9, 7.6 ± 2.8, and 10.2 ± 3.1, *P *< 0.001) were observed in women with more frozen blastocysts. No statistically significant difference was noted in FSH, BMI, the proportion of primary infertility, and endometrial thickness among the groups. Ovarian hyperstimulation syndrome (OHSS) rates did not differ significantly, which were 1.8%, 1.9%, and 2.1% in the three groups, respectively.

### Live birth rates by the number of frozen blastocysts

Figure 1 depicts live birth rates with 95% CIs by the number of frozen blastocysts. The number of patients with blastocysts of 0, 1, 2, 3, 4, and ≥ 5 was 1561 (18.0%), 1386 (16.0%), 1557 (17.9%), 1160 (13.4%), 1053 (12.1%), and 1959 (22.6%), respectively. The live birth rate in patients with 0 blastocyst frozen was 36.0% and increased to 46.2% when patients had 1 blastocyst (*P *< 0.001). But no significant increase was appeared with increasing number of frozen blastocysts.

### Clinical outcomes among the three groups

Live birth rates in patients with ≥ 3 and 1–2 frozen blastocysts were 47.6% and 46.1%, respectively, which were significantly higher than that in patients without blastocyst (36.0%) (Table 2). The clinical pregnancy rate in group 3 was 65.1%, which was also significantly higher than the other two groups (47.0% and 59.2%). The similar tendency of live birth and pregnancy rates were also revealed after being adjusted for female age, years of infertility, FSH, AFC, BMI, type of infertility, the number of embryos transferred, endometrial thickness, and type of treatment (IVF or ICSI). The implantation rates were 35.5%, 47.6%, and 56.0% in the three groups, respectively (*P *< 0.001).

**Table 2:** Clinical Outcomes

	Number of frozen blastocysts			
	**0**	**1–2**	** ≥ 3**	*P*
Live birth rate (%)	36.0 (562/1561)	46.1 (1357/2943)	47.6 ^a^ (1987/4172)	< 0.001
Adjusted ^b^ live birth rate	0.35	0.47	0.50	< 0.001^C^
Clinical pregnancy rate (%)	47.0 (733/1561)	59.2 (1743/2943)	65.1 (2716/4172)	< 0.001
Adjusted ^b^ clinical pregnancy rate	0.47	0.59	0.66	< 0.001^C^
Implantation rate (%)	35.5 (854/2409)	47.6 (2110/4430)	56.0 (3131/5588)	< 0.001
Miscarriage rate (%)	23.3 ^a^ (171/733)	22.1 (386/1743)	26.8 (729/2716)	0.001

^a^*P *> .05 vs group 2 (number of frozen blastocysts = 1–2)

^b^ Values are marginal means (95%CIs) adjusted for female age, years of infertility, FSH, AFC, BMI, type of infertility, the number of embryos transferred, endometrial thickness, and type of treatment (IVF or ICSI)

^c^ Values are obtained from x^2^ test (based on the marginal mean of estimation)

### Multivariable logistic regression models

The OR (95% CI) for live birth rate is listed in Table 3, showing that in patients with ≥ 10 oocytes retrieved, the independent influencing factors were the maternal age, AFC, endometrial thickness, and the number of embryos transferred. Compared with that in the reference group, 0 blastocyst frozen was associated with a lower rate of live birth (the adjusted ORs: 0.526, 95% CI: 0.469, 0.612). However, the difference was not found between groups of 1–2 and ≥ 3 frozen blastocysts.

**Table 3:** Logistic regression model of live birth

	**OR**	**95%CI**	**Wald x**^2^	***P***
**Number of frozen blastocysts**				
0	0.526	(0.469, 0.612)	84.392	< 0.001
1–2	0.867	(0.781, 0.963)	7.095	0.008
≥ 3	Reference			
Age (years)	0.952	(0.939, 0.965)	49.053	< 0.001
Years of infertility	1.002	(0.982, 1.024)	0.053	0.817
FSH	0.998	(0.971, 1.025)	0.028	0.867
AFC	1.020	(1.020, 1.028)	22.642	< 0.001
BMI	1.001	(0.986, 1.017)	0.020	0.887
Primary	0.960	(0.865, 1.066)	0.575	0.448
Secondary	Reference			
1 embryo transferred	0.500	(0.454, 0.550)	203.263	< 0.001
2 embryos transferred	Reference			
Endometrial thickness	1.050	(1.031, 1.069)	28.094	< 0.001
IVF	0.986	(0.894, 1.089)	0.074	0.786
ICSI	Reference			

*FSH* Follicle stimulating hormone, *AFC* Antral follicle count, *BMI* Body mass index, *IVF* In vitro fertilization and embryo transfer, *ICSI* Intracytoplasmic sperm injection

## Discussion

The central finding of this retrospective study is that the number of frozen blastocysts is a strong indicator of live birth rates in fresh embryo transfer cycles. Whether patients have utilizable blastocysts frozen or not is associated with the pregnancy outcomes of the cleavage stage embryos within an embryo cohort. In addition, if the number of blastocysts was not 0, the clinical outcomes did not differ from different numbers.

While conventional morphological grading is helpful for determining the optimal quality of embryos to transfer, this first-line assessment has limited value in predicting the live birth [12, 13]. A study of 819 cycles advocated that quality of supernumerary embryos was positively associated with the implantation rate of transferred embryos in patients with ≥ 3 blastocysts available on day 5 [10]. But this association was attenuated when controlling for the quality of transferred embryos, which restricted its clinical application in prophesying pregnancy achievement. Embryo quality varied widely from different stages due to different culture environment and as pointed out by Petracco [14]. However, the number of frozen blastocysts is another parameter that should not be ignored. The human embryo undergoes multiple procedures in vitro as it develops and differentiates from a fertilized oocyte to the blastocyst stage. Blastocyst development by extended culture allows the embryos to ‘select’ themselves [15]. Successful growth to the blastocyst stage is a reflection of the developmental competence of their former cleavage stage embryos. Thus, we hypothesized that the number of frozen blastocysts was a strong predictor of the quality of the transferred cleavage stage embryos in the entire cohort.

The aim of this paper is to highlight the importance of the number of frozen blastocysts on predicting live birth rates in fresh embryo transfer cycles. Considering the statistical influence, only patients with optimal ovarian response were chosen in our study since patients with less oocytes retrieved might have few available embryos for freezing. By graphing the rate of live birth by the number of frozen blastocysts (Fig. 1), live birth rates in patients with frozen blastocysts were significantly higher than that in patients without blastocyst. The observed increase of live birth rate associated with the number of blastocysts frozen is consistent with prior studies which displayed that patients with live birth outcome had more utilizable blastocysts than patients without a live offspring [16–18]. Very similar findings were reported that women who were more likely to have blastocysts by day 5 to 6 had better average quality of embryos by day 3 [19].

Previous literature has explored the contributing factors predicting the success of IVF cycles, in which some are the most widely known such as the female age, AFC, and endometrial thickness [20–24]. Because all patients had optimal ovarian response according to the inclusion criteria, their average age were similar among groups (*P *= 0.077, Table 1), which minimized bias in this study. As expected, more AFC, another contributing factor, were observed in group 3 than the other two groups. But we did not see the clinical significant differences between 15 and 16 AFC especially in all patients with optimal ovarian response. Endometrial thickness was also similar among the three groups (12.3 vs. 12.0 vs. 12.0, respectively). In particular, the number of embryos transferred was the essential factor of the live birth. The lower proportion of patients with two embryos transferred could explain why implantation rates for those with ≥ 3 frozen blastocysts were 56.0% but the live birth rate was only 47.6% in Table 2. 34.0% (1416/4172) of these patients had two embryos transferred while in groups with 0 and 1–2 frozen blastocysts, around 50% patients had two embryos transferred. In line with this, we found that the number of blastocysts frozen, which summarized the average developmental function of the collective embryos within a cohort, was independent of successful pregnancy in optimal ovarian responders even after being adjusted for the confounding factors. Compared with that in the reference group, 0 blastocyst frozen was associated with a lower rate of live birth (the adjusted ORs: 0.526, 95% CI: 0.469, 0.612) (Table 3).

The number of frozen blastocysts was in essence manifestations of the average quality of embryos in a cohort. It was acknowledged that not all embryos would reach the blastocyst stage and be cryopreserved because of longer culture in vitro. In our study, 18.0% patients (1561/8676) did not have any blastocyst for cryopreservation after day 3 embryo transfer though they obtained ≥ 10 oocytes. The reasons might be explained by the number of 2PN, which were 5.7 ± 2.9, 7.6 ± 2.8, and 10.2 ± 3.1 in the three groups, respectively (Table 1). The less 2PN led to the less available embryos the patients in the group 1 might have. There is now convincing evidence to suggest that the intrinsic quality of the oocyte is the key factor determining the number of oocytes attaining of the blastocyst stage [25–28], which accorded with the findings of our study. The average blastocyst formation rate of group 1 (patients without blastocyst frozen) was 30.8%, which was significant lower than the other two groups (44.8% and 70.1%). These may provide explanations on the associations between the number of blastocysts and live birth rates of the transferred fresh embryos.

Interesting, the results of our study showed that the rate of live birth did not differ among different numbers of frozen blastocysts. Having frozen blastocysts (if not = 0), not quantity, was enough to be an indicator of pregnancy success. The mean number of oocytes retrieved was 13.0 and 13.5 in group 1 (0 frozen blastocyst) and group 2 (1–2 frozen blastocysts) respectively (Table 1), but the extremely different rate of live birth was demonstrated in the two groups (Table 2). This can help us better understand why the live birth rate does not continue to boost as expected after the number of oocytes retrieved reaches 16. A large sample study of 400,135 IVF cycles made a point that live birth rates rose with an increasing number of eggs up to 15, and then plateaued [29].

This finding is relevant for clinicians not only in medical practice, but also in future clinical research design. For example, the question of whether to adopt ‘freeze-all embryos’ strategy is very topical today. Attention has been dedicated to controlling ovarian function or oocyte quality between fresh embryo transfer and frozen-thawed embryo transfer cycles when assessing whether ‘freeze all’ strategy is beneficial. Meanwhile, it is advised to control the number of frozen blastocysts which is another non-ignorable factor of embryo quality in a cohort according to the results of our study.

Limitations of our study include the study design, which is a retrospective research from a single medical center. However, the large sample size, use of a multivariate regression model for a wide array of possible confounding factors, and marginal means of adjusted live birth rate rendered the conclusion relatively reliable. Furthermore, it’s a pity that the data of the quality of blastocysts cannot be obtained at present, which can potentially maximize cumulative live birth rates. Last but not least, even the number of blastocysts frozen was an independent marker of predicting live birth, the fresh D3 embryo transfer was followed by blastocyst formation. This might limit the clinical application of this data in embryo selection.

## Conclusion

In patients with optimal ovarian response that retrieved ≥ 10 oocytes, fresh embryos transfer followed by having blastocysts frozen is a strong indicator of the production of live birth, but the number of frozen blastocysts (if not = 0) has limited value in predicting live birth rates. Our observations highlight the importance of the number of frozen blastocysts, which not only provide informed evidence to clinicians in medical practice, but should also be considered in future clinical research on the success of assisted reproduction.

## Data Availability

The datasets used and/or analysed during the current study are available from the corresponding author on reasonable request.
